# Asbestos modulates thioredoxin-thioredoxin interacting protein interaction to regulate inflammasome activation

**DOI:** 10.1186/1743-8977-11-24

**Published:** 2014-05-20

**Authors:** Joyce K Thompson, Catherine M Westbom, Maximilian B MacPherson, Brooke T Mossman, Nicholas H Heintz, Page Spiess, Arti Shukla

**Affiliations:** 1 Department of Pathology, University of Vermont, College of Medicine, Burlington, VT 05405, USA

**Keywords:** Asbestos, Malignant mesothelioma, Thioredoxin, Thioredoxin interacting protein, Inflammasomes

## Abstract

**Background:**

Asbestos exposure is related to various diseases including asbestosis and malignant mesothelioma (MM). Among the pathogenic mechanisms proposed by which asbestos can cause diseases involving epithelial and mesothelial cells, the most widely accepted one is the generation of reactive oxygen species and/or depletion of antioxidants like glutathione. It has also been demonstrated that asbestos can induce inflammation, perhaps due to activation of inflammasomes.

**Methods:**

The oxidation state of thioredoxin was analyzed by redox Western blot analysis and ROS generation was assessed spectrophotometrically as a read-out of solubilized formazan produced by the reduction of nitrotetrazolium blue (NTB) by superoxide. Quantitative real time PCR was used to assess changes in gene transcription.

**Results:**

Here we demonstrate that crocidolite asbestos fibers oxidize the pool of the antioxidant, Thioredoxin-1 (Trx1), which results in release of Thioredoxin Interacting Protein (TXNIP) and subsequent activation of inflammasomes in human mesothelial cells. Exposure to crocidolite asbestos resulted in the depletion of reduced Trx1 in human peritoneal mesothelial (LP9/hTERT) cells. Pretreatment with the antioxidant dehydroascorbic acid (a reactive oxygen species (ROS) scavenger) reduced the level of crocidolite asbestos-induced Trx1 oxidation as well as the depletion of reduced Trx1. Increasing Trx1 expression levels using a Trx1 over-expression vector, reduced the extent of Trx1 oxidation and generation of ROS by crocidolite asbestos, and increased cell survival. In addition, knockdown of TXNIP expression by siRNA attenuated crocidolite asbestos-induced activation of the inflammasome.

**Conclusion:**

Our novel findings suggest that extensive Trx1 oxidation and TXNIP dissociation may be one of the mechanisms by which crocidolite asbestos activates the inflammasome and helps in development of MM.

## Background

Malignant mesothelioma (MM) is a deadly cancer arising from the mesothelium and its etiology usually involves asbestos exposure [1]. MM is a very invasive and aggressive disease that is chemo-resistant to most of the standard chemotherapeutic agents. Patients with MM typically have a poor prognosis with a life expectancy of about 8-12 months after diagnosis [2]. Efforts at understanding how asbestos exposure leads to the development of MM and other malignancies have not definitively determined how exposure leads to the formation and progression of this unusual neoplasm. Studies have, however, shown that apoptosis followed by compensatory proliferation and chronic inflammation induced by asbestos fibers play a major role in disease progression [3-5]. Chronic inflammation induced by asbestos exposure is believed to be involved in the pathogenic process that leads to asbestos related diseases like MM [6,7]. Recent work from our group has demonstrated that asbestos-induced inflammation in mesothelial cells and machrophages could, in part, be mediated by activation of the inflammasome, a protein complex involved in the processing of cytokines [8]. The exact mechanism by which asbestos activates the inflammasome is not completely understood, but reactive oxygen species (ROS) are believed to play a role [5]. It has also been reported that a redox-regulated protein, thioredoxin interacting protein (TXNIP) can bind and activate the Nod-like receptor family pyrin domain containing 3 (NLRP3) inflammasome [9]. ROS induced in response to crocidolite asbestos exposure have been shown to initially deplete intracellular levels of reduced glutathione [10,11], but the effect of crocidolite asbestos on another major cellular antioxidant, thioredoxin (Trx1) is unknown.

Thioredoxin is a small ubiquitously expressed redox active protein that is important for maintaining the reducing milieu of the cell, in part by reducing protein disulfide bonds that occur in response to oxidative processes. During reduction of disulfide bonds Trx1 itself becomes oxidized and in turn reduced by thioredoxin reductase (TR) using electrons from reduced nicotinamide adenine dinucleotide phosphate (NADPH) [12]. Trx1 is inhibited by thioredoxin interacting protein (TXNIP) via a redox-dependent interaction [13,14]. TXNIP is only capable of binding to and inhibiting Trx1 in its reduced state [9,14]. In response to oxidative insults, TXNIP has been shown to bind to and activate the NLRP3 inflammasome [9]. Based on these observations and the capacity for crocidolite asbestos fibers to generate ROS intra- and extracellularly, we hypothesized that crocidolite asbestos-induced ROS generation will oxidize Trx1 causing its dissociation from TXNIP. As a result of this dissociation, TXNIP would be free to bind to, and activate, the NLRP3 inflammasome. Here, we show for the first time, that crocidolite asbestos exposure leads to the irreversible oxidation of Trx1 and depletes reduced Trx1 levels in LP9/hTERT cells. We also show that over-expression of Trx1 reduces levels of crocidolite asbestos-induced ROS. Our results indicate that oxidation of Trx1 by crocidolite asbestos results in dissociation of TXNIP and subsequent activation of inflammasomes, as knockdown of TXNIP by siRNA partially reduced crocidolite asbestos-induced inflammasome activation as indicated by a reduction in caspase-1 activation.

## Materials and methods

Human LP9 mesothelial cells, an hTERT-immortalized cell line that phenotypically and functionally resembles normal human mesothelial cells, were obtained from Dr. James Rheinwald (Brigham and Women’s Hospital, Harvard University, Boston, MA). All cells were incubated at 37°C in 5% CO_2_ and grown to 80–90% confluency as described previously [15]. The physical and chemical characterization of the National Institute on Environmental Health Sciences (NIEHS) reference sample of crocidolite asbestos has been reported previously [16]. The NIEHS chrysotile reference sample was used for asbestos fiber comparisons. After sterilization under UV light overnight, particulates were suspended in Hank’s balanced salt solution (HBSS) at 1 mg/ml, sonicated for 15 min in a water bath sonicator, and triturated five to ten times through a 22-gauge needle. A volume of this suspension was added to cells in medium to achieve the desired final concentration of 75 × 10^6^ μm^2^/cm^2^ dish surface area, a concentration known to cause apoptosis and compensatory proliferation of surrounding rat pleural mesothelial and murine alveolar type II epithelial cells [3,17]. Glass beads (Polysciences Inc, Warrington, PA) were used as a non-pathogenic particle control. Dehydroascorbic acid (DHA) and 2,4-Dinitro-1-chlorobenzene (DNCB) were purchased from Sigma (St. Louis, MO). Trx1 expression vector (pCMV-SPORT6) and pcDNA (empty vector control) used for over-expression studies were obtained from Dr. Nicholas Heintz.

### Western blot analyses

Cells grown in 60 mm culture dishes were washed 3× with ice-cold phosphate buffered-saline (PBS), collected in lysis buffer (20 mM Tris pH 7.6, 1% Triton X-100, 137 mM NaCl, 2 mM EDTA, 1 mM Na_3_O_4_V, 10 mM NaF, 1 mM DTT, 1 mM phenylmethylsulfonyl fluoride, 10 μg/ml leupeptin, and 10 μg/ml aprotinin) and incubated on ice for 30 min. Lysates were centrifuged at 14,000 rpm for 15 min at 4°C. Supernatants were collected, and protein concentrations were determined using the Bradford assay (Bio-Rad, Richmond, CA). Cell lysates (40 μg protein per lane) were resolved by one-dimensional SDS-PAGE and transferred to nitrocellulose membranes according to standard procedures. Equal loading of protein was verified by β-actin (Abcam, Cambridge, MA). Membranes were washed in Tris-buffered saline (TBS), and blocked for 2 h with TBS-Tween (TBST) containing 10% nonfat milk, then incubated with rabbit anti-human Trx1 antibody (Abcam, Cambridge, MA) at 1:5000 dilution in TBST containing 5% nonfat milk and 0.01% sodium azide overnight at 4°C. Membranes were washed four times with TBST for 15 min each time prior to incubation with secondary antibody. Western blots shown are representative blots with their accompanying densitometric analysis.

### Determination of the redox state of thioredoxin

The redox state of thioredoxin in response to crocidolite asbestos exposure was determined using the redox Western blot method as previously described [18]. Briefly, cells were lysed in 6 M guanidine HCl buffer (6 M Guanidine HCl; 50 mM Tris/Cl pH 8.3; 3 mM EDTA, 0.5% Triton X – 100; 10 μg/ml aprotonin and 10 μg/ml leupeptin) containing 50 mM iodoacetic acid (IAA) for alkylation of the thiol groups of thioredoxin. Cells were incubated in lysis buffer at 37°C for 30 min in the dark. Excess IAA was removed by spinning lysates on Amicon centrifugal concentrating columns with a 10,000 nominal molecular weight limit (NMWL) (EMD Millipore, Billerica, MA). In order to exchange the IAA containing buffer, the lysates were washed 3 times with a HEPES buffer at pH 7.4 and the concentrated lysates were collected in fresh collection tubes by inverting the columns in the tubes. After protein determination by the Bradford method (Bio-Rad, Richmond CA), 40 μg of protein was loaded onto a 15% non–reducing native polyacrylamide gel using a 1× Tris Glycine (pH 8.8) running buffer. The electrophoresis was carried out at 75 V for approximately 3.5 h. The redox gel was then washed in 50 mM Tris (pH 8.3) for 5 min and then equilibrated in 1× transfer buffer by washing in buffer 3 times for 5 min each. Thereafter, the proteins were transferred onto a nitrocellulose membrane by wet transfer at 100 V for 2 h. The nitrocellulose membrane was then blocked with 10% milk in 1× Tris buffered saline with Tween 20 (TBS-T) for 2 h at room temperature (RT) and incubated in anti-Trx1 primary antibody (1:5000 in 5% milk/TBS-T, Abcam, Cambridge, MA) overnight at 4°C. Goat anti–rabbit secondary antibody conjugated to horseradish peroxidase (1:2000 in 1X TBS-T, Jackson ImmunoResearch Laboratories Inc. West Grove, PA) was used and visualization was done by enhanced chemiluminescence reagents (Amersham Pharmacia Biotech, Piscataway, NJ) on X-ray film. Blots were quantified using Quantity One software (Bio-Rad, Richmond, CA). Distribution of redox states of Trx1 was determined as band intensity of reduced or oxidized Trx/(reduced + semi-oxidized + fully oxidized) as described by Watson et al., (2003) [18].

### Real-time quantitative PCR (qRT-PCR)

Total RNA was prepared using an RNeasy plus mini kit according to the manufacturer’s protocol (Qiagen, Valencia, CA) as described previously [15]. Total RNA (1 μg) was reverse-transcribed with random primers using the Promega AMV Reverse Transcriptase kit (Promega, Madison, WI) according to the recommendations of the manufacturer. To quantify gene expression, the cDNA was amplified by TaqMan Real Time Q-PCR using the 7900HT SequencePrism Detector (Applied Biosystems, Foster City, CA). Duplicate assays were performed with RNA samples isolated from at least four independent experiments. Fold changes in gene expression were calculated using the delta-delta Ct method. The values obtained from cDNA and hypoxanthine phosphoribosyl transferase (*HPRT*) controls helped determine relative gene expression levels for the gene locus investigated. The Assay on Demand primers and probes used were purchased from Applied Biosystems.

Since exposure of cells to chrysotile asbestos had no effect on the oxidation state of Trx1, all subsequent experiments were performed using crocidolite asbestos. All references to asbestos relate to crocidolite asbestos unless otherwise specified.

### Dehydroascorbic acid (DHA) pretreatment

In order to investigate whether the ROS generated by asbestos exposure was responsible for the extensive oxidation of thioredoxin, cells where pretreated with the ROS quencher, dehydroascorbic acid (1 mM) for 1 h before exposure to asbestos for 8 h.

### Antioxidant pretreatment of cells by N-acetylcysteine (NAC)

To investigate the role of the asbestos-induced ROS on inflammasome activation, cells were pretreated with 2 mM NAC for 20 h as previously described (Shukla et al., 2004). Briefly, cells were grown to 90% confluency in 60 mm dishes and serum starved by replacing complete medium with 0.5% fetal bovine serum (FBS) supplemented medium for 6 h prior to addition of NAC diluted in HBSS at pH 7.4 (cells were maintained in reducing medium for the entire duration of NAC pretreatment). After pretreatment with NAC, cells were exposed to asbestos for 48 h. Thereafter, inflammasome priming was assessed by qRT-PCR of NLRP3 transcript levels while activation was analyzed by Western blot analysis of caspase-1, p20 fragment.

### Treatment with 2,4-Dinitro-1-chlorobenzene DNCB

To obtain a final concentration of 10 μM in culture, 2,4-Dinitro-1-chlorobenzene (DNCB) (an irreversible alkylating inhibitor of TR) was dissolved in DMSO with a final DMSO content of 0.2% which was determined to be non-cytotoxic in previous experiments. For all experiments, cells were pretreated with DNCB for 1 h prior to exposure to asbestos.

### Lactate dehydrogenase (LDH) activity assay

To determine the cytotoxic effects of DNCB and asbestos exposure on LP9 cells, an LDH assay was performed using the LDH kit from Promega, (Madison WI) according to the manufacturer’s direction. Briefly, 50 μl of media were collected from each dish in triplicate into a 96 well plate using cells lysed by the addition of 0.9% Triton X-100 as a positive control. To each of these wells, 50 μl of LDH substrate buffer was added and the reaction was incubated on a rotary shaker at room temperature for 30 min in the dark. Thereafter, the reaction was terminated by the addition of 50 μl stop buffer to each well. Any bubbles present were broken with a hypodermic needle and the plate was read spectrophotometrically at 490 nm in a 96 well plate reader. Cytotoxicity was expressed as a percentage of LDH released relative to the lysis control.

### Detection and quantitation of apoptosis

To determine whether modulation of Trx1 protein levels and oxidation state altered cell death in human mesothelial cells, detection of apoptosis was performed using the ApoStain technique as described previously (Shukla *et al*., 2003). In brief, cells were grown on glass cover slips and exposed to asbestos with or without DNCB (10 μM) for 8 h. The cover slips were then processed to determine the numbers of apoptotic cells and total cell numbers per field. Five random fields were evaluated at a magnification of 400× on each cover slip.

### Assessment of pyroptosis by asbestos

Since asbestos causes inflammasome activation, as measured by caspase-1 activation [4], we were interested in learning if asbestos-induced cell death may be due in part to pyroptosis (caspase-1 dependent cell death) or not. For this purpose we pretreated LP9 cells with a specific caspase-1 inhibitor (40 μM Caspase-1 inhibitor VI (zYVADfmk), EMD Biosciences, Billerica, CA) for 1 h and subsequently with asbestos for 24 h. The number of viable cells was determined after trypsinization and counting of cells on a hemocytometer at the end of the experiment. Media supernatants were also analyzed for the levels of the p20 subunit of active caspase 1 by Western blot analysis.

### Transfection procedures

#### Trx1 over- expression

Cells at 90% confluence were transfected with pcDNA (empty vector control, 4 μg DNA per 60 mm dish) and human Trx1 over expression vector (pCMV-SPORT6, 4 μg DNA per 60 mm dish) using Lipofectamine 2000 (10 μl) (Life Technologies, Grand Island, NY), following the manufacturer’s protocol. The efficiency of Trx1 protein over expression was determined by qRT-PCR after 48 and 72 h.

### Knockdown of TXNIP

LP9 cells that were 90% confluent were transfected with either ON-TARGET plus smart pool human TXNIP siRNA (siTXNIP) or ON-TARGET plus non-targeting siRNA (siControl) from Dharmacon (Fisher Scientific, Pittsburgh, PA) using Lipofectamine 2000 (Life Technologies, Grand Island, NY) diluted in a final volume of 500 μl Optimem medium (Life Technologies, Grand Island, NY), as previously described [15]. All siRNA were reconstituted to 20 μM before transfection and stored at -20°C until use. The magnitude of TXNIP knockdown was assessed by qRT-PCR.

To confirm observations of the role of TXNIP in inflammasome activation, a human mesothelioma cell line (HMESO) in which the extracellular signal regulated kinase 2 had been stably knocked down (shERK2) was used. These cells have been previously reported (GSE21750) to have down-regulated expression of TXNIP (several fold). To activate the inflammasome, shERK2 HMESO cells and corresponding control cells stably transfected with non-targeting shRNA (shCon) were treated with 5 μM doxorubicin (Dox) as previously described [19]. After 48 h of treatment the shERK2 and shCon HMESO cells, treated with or without Dox, medium supernatants were harvested by centrifuging medium at 300 × g for 7 min in the cold (4°C) to remove cellular debris. The resulting supernatants, stored in 1 ml aliquots, were concentrated using Amicon centrifugal concentrating columns with a 10,000 nominal molecular weight limit (NMWL) (EMD Millipore, Billerica, MA). 4× sample buffer was added to the concentrated supernatant to a final concentration of 1× before being electrophoresed at 100 V for 2 h on a 15% SDS PAGE gel. Immunobloting was performed as described above with caspase-1 p20 antibody (Cell Signaling, Danvers MA) to detect caspase-1 activation.

### Statistical analysis

All data were analyzed by one-way ANOVA compared with the respective control group and a Neuman-Keuls post test for multiple comparisons or the Student’s ‘t’test. Results are presented as the mean ± SEM. All experiments were repeated at least twice or more. Comparisons with a p value ≤ 0.05 were considered statistically significant. Statistical analyses were conducted using Graph Pad Prism v6 software.

## Results

### The effects of crocidolite asbestos exposure on thioredoxin 1 (Trx1) expression in human mesothelial cells

**Figure 1:**
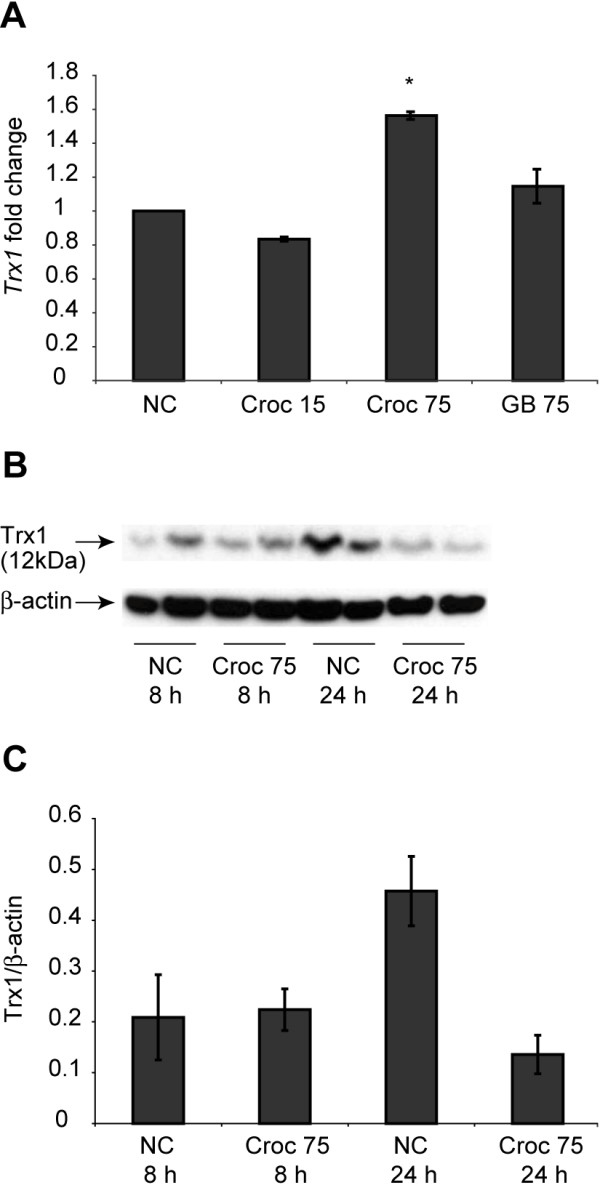
**Crocidolite asbestos exposure modulates Trx1 levels in mesothelial cells. (A)** LP9 cells were exposed to crocidolite asbestos at 75 × 10^6^ μm^2^/cm^2^ for 8 and 24 h. Glass beads (GB) at the same surface area concentration were used as an inert particulate control. RNA was extracted from the samples and used to prepare cDNA, which was quantified by qRT-PCR (*p < 0.05 compared to untreated controls (NC)). **(B)** Western blot analysis of LP9 cells for total thioredoxin protein after exposure to crocidolite asbestos, β-actin was used as a loading control. **(C)** densitometric analysis of **(B)** using Quantity One software (n =2 per group) .

To determine the effect of crocidolite asbestos exposure on the expression levels of Trx1, LP9/h-TERT cells were exposed to 75 × 10^6^ μm^2^/cm^2^ (or 5 μg/cm^2^ ) crocidolite for 8 and 24 h and RNA was extracted. Analysis of fold changes in mRNA levels for Trx1 by qRT-PCR revealed a 1.6 fold increase in Trx1 mRNA levels after 24 h of crocidolite asbestos exposure as compared to controls (*p < 0.05) (Figure 1A). An assessment of the total Trx1 protein levels after crocidolite asbestos exposure showed a decrease in protein levels after 24 h (Figure 1B & C). In contrast, glass beads (which were used as a negative control) and crocidolite asbestos at surface area coverage of 15 × 10^6^ μm^2^/cm^2^ (or 1 μg/cm^2^) did not cause a significant increase in mRNA levels at 24 h as compared to untreated controls.

### The oxidation state of thioredoxin 1 (Trx1) after crocidolite exposure

**Figure 2:**
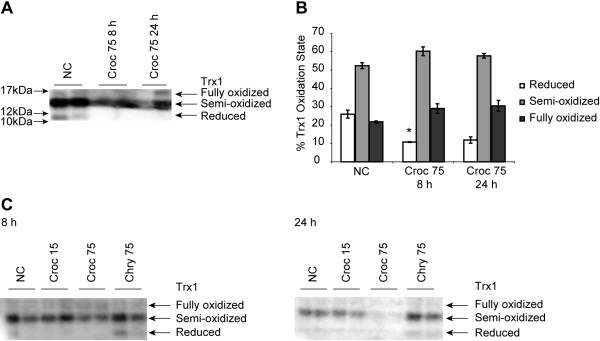
**Crocidolite asbestos exposure causes oxidation of Trx1 in human mesothelial cells. (A)** LP9/h-TERT cells were exposed to crocidolite asbestos at 75 × 10^6^ μm^2^/cm^2^ for 8 and 24 h and 40 μg of protein was run on a 15% native gel and a redox Western blot analysis was performed as described in the Methods section on the immobilized proteins. **(B)** Quantitation of the blot in **(A)**. **(C)** Cells were exposed to two concentrations of crocidolite asbestos (15 × 10^6^ μm^2^/cm^2^ and 75 × 10^6^ μm^2^/cm^2^), as well as chrysotile asbestos (75 × 10^6^ μm^2^/cm^2^) and a redox Western analysis was performed on the lysates (*p < 0.05 compared to null controls (n = 2 per group).

LP9 cells exposed to 75 × 10^6^ μm^2^/cm^2^ crocidolite asbestos for 8 and 24 h showed a decrease in the proportion of reduced Trx1 as assessed by redox Western analysis as compared to untreated controls (Figure 2A). This decrease in the level of reduced thioredoxin was not observed in cells treated with sodium arsenite, which has been shown previously to oxidize Trx1 in a different cell type [20]. In addition, the total amounts of Trx1 observed in crocidolite asbestos exposed cells appeared to be lower than the levels in the other treatment groups. A third band that represented fully oxidized thioredoxin was present in cells exposed to either crocidolite asbestos or arsenite, but was not observed in lysates from the untreated controls. To determine whether the oxidation of thioredoxin by crocidolite was specific to crocidolite asbestos alone, LP9 cells were also exposed to chrysotile asbestos and glass beads. As assessed by redox Westerns, oxidation of thioredoxin was specific to the higher concentration (75 × 10^6^ μm^2^/cm^2^) of crocidolite asbestos alone (Figure 2C). Levels of reduced and semi-oxidized Trx1 remained the same at 8 and 24 h in response to chrysotile and glass bead exposure.

### Inhibition of thioredoxin reductase (TR) by dinitrochlorobenzene (DNCB) and pretreatment with dehydroascobic acid

**Figure 3:**
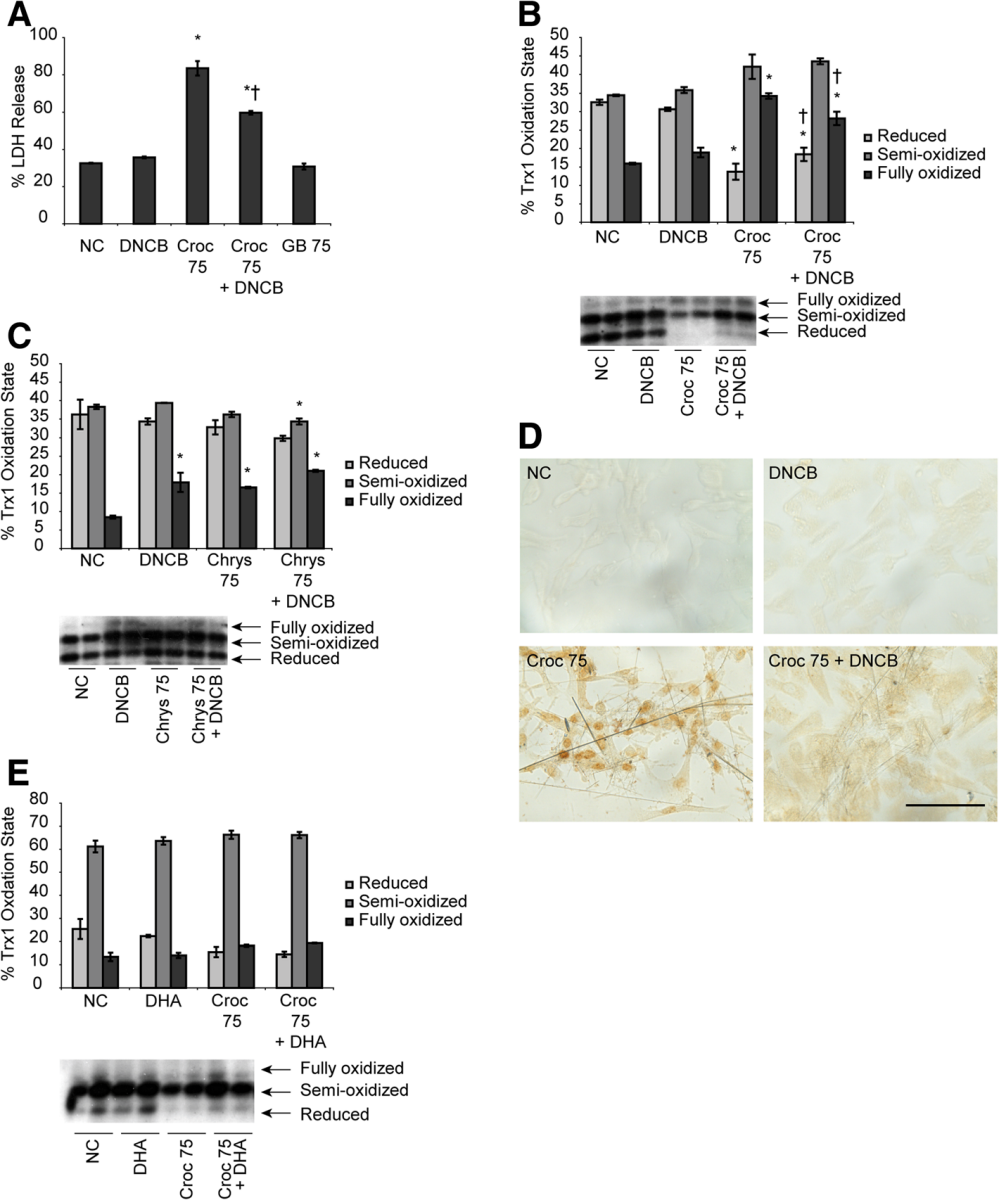
**Inhibition of thioredoxin reductase by DNCB and pretreatment of cells with DHA rescues asbestos-induced oxidation of Trx1. (A)** LDH assay to assess lytic cell death after pretreatment of LP9 cells with DNCB and exposure to asbestos (data is presented as a percentage of the lytic control). **(B)** Effect of DNCB on asbestos-induced Trx1 oxidation. Cells were pretreated with 10 μM DNCB for an hour and then exposed to asbestos for 8 h. Cell lysates were then derivatized with IAA and analyzed for oxidation of Trx1 by redox Western blot and densitometry of redox Western analysis of Trx1 oxidation state was performed. **(C)** Effect of DNCB pretreatment on chrysotile asbestos-induced oxidation of Trx1 **(D)** Analysis of apoptosis in response to DNCB pretreatment and asbestos exposure for 8 h as measured by Apostain technique. **(E)** LP9 cells were pretreated with 1 mM DHA for an hour and exposed to asbestos for 8 h. Thereafter, the oxidation state of Trx1 was assessed by redox Western blot analysis (*p < 0.05 compared to null controls; †p < 0.05 compared to crocidolite asbestos exposure alone (Croc 75 n = 2 per group).

To determine whether inhibition of TR by DNCB would increase asbestos-induced oxidation of Trx1, LP9 cells were pretreated with 10 μM DNCB and exposed to 75 × 10^6^ μm^2^/cm^2^ crocidolite asbestos for 8 and 24 h. A lactate dehydrogenase (LDH) assay performed on the medium from exposed cells showed that LDH levels were reduced in cells pretreated with DNCB when compared with cells exposed to asbestos alone (Figure 3A). In addition, redox Western blot analysis of cell lysates indicated that the oxidation of Trx1 by asbestos was ameliorated when cells were pretreated with DNCB before exposure to crocidolite asbestos (Figure 3B). On the contrary, cells exposed to chrysotile asbestos with and without DNCB showed no changes in the redox states of Trx1 when compared to controls (Figure 3C). An Apostain assay to detect chromatin condensation and single strand breaks in nuclear DNA as a measure of early apoptotic events was also conducted to determine the effects of pretreatment with DNCB on asbestos-induced apoptosis. Apostain revealed that cells pretreated with DNCB were protected from apoptosis after 8 h of exposure to crocidolite asbestos (Figure 3D). On the other hand, pretreatment of LP9 cells with DHA before exposure to asbestos slightly increased the amount of reduced thioredoxin in cells compared to asbestos exposure alone (Figure 3E). However, there was no significant increase when compared to control or asbestos exposure alone.

### ROS generation in response to asbestos is modulated by Trx1 in LP9 cells

**Figure 4:**
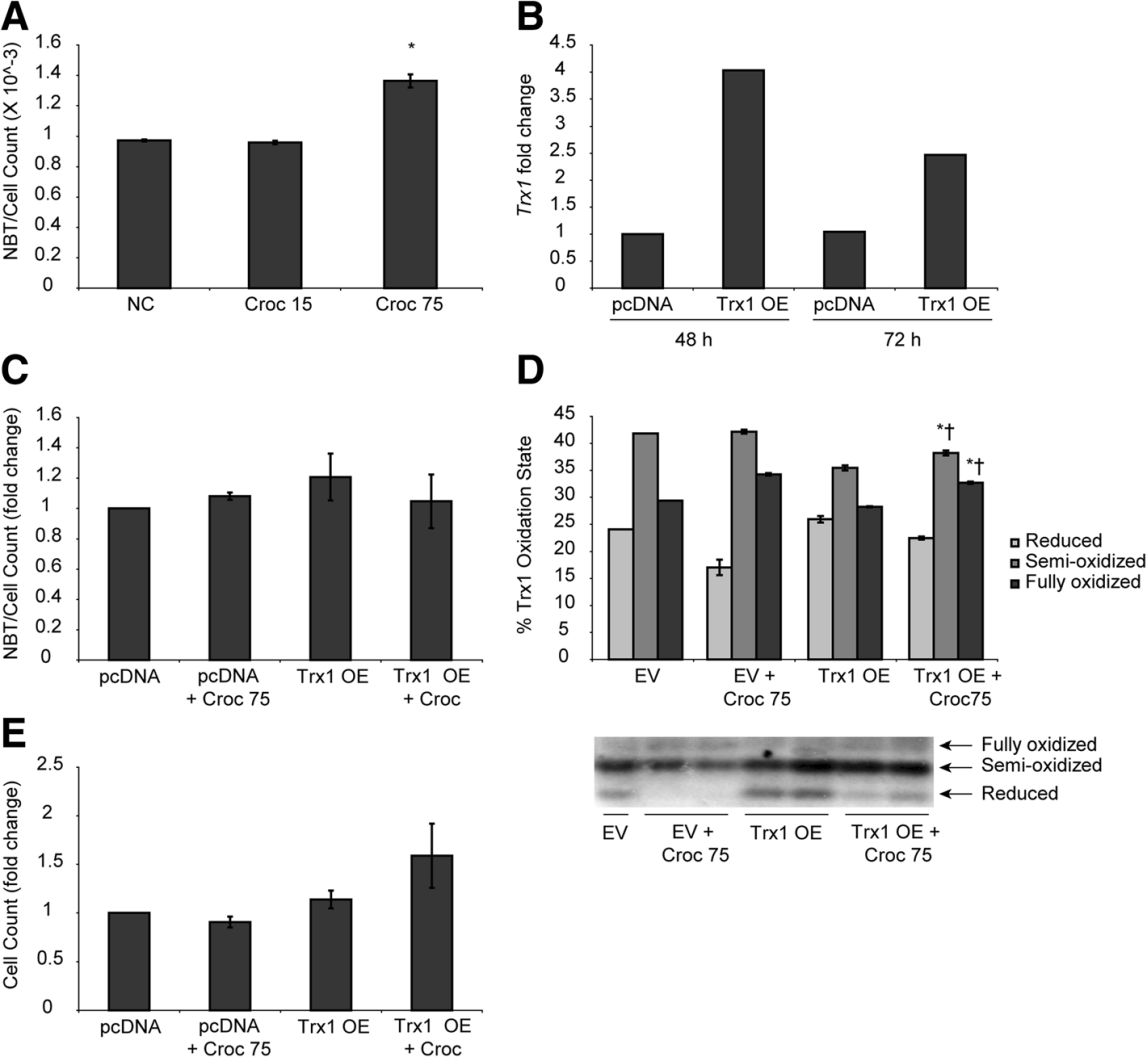
**Over-expression of Trx1 increases cell survival and ameliorates asbestos-induced ROS generation in LP9 cells. (A)** LP9 cells were exposed to two doses of crocidolite asbestos (15 × 10^6^ μm^2^/cm^2^ and 75 × 10^6^ μm^2^/cm^2^) for 24 h and incubated with NBT for 45 min at 37°C. The absorbance of the solubilized formazan formed after incubation with NBT was then read at 630 nm to determine ROS levels after asbestos exposure. **(B)** Over-expression of Trx1 in LP9 cells using the pCMV-SPORT6 plasmid was confirmed by qRT-PCR 48 and 72 h after transfection. **(C)** Trx1 transfected cells were exposed to crocidolite asbestos for 2 h and incubated with NBT for 45 min to determine ROS levels. Solubilized formazan was measured spetrophotometrically at 630 nm on a plate reader. **(D)** Analysis of the oxidation state of Trx1 after asbestos exposure of Trx1 over-expressing LP9 cells was determined by redox Western blot analysis and densitometry of the blot was performed (n = 2 per group). **(E)** LP9 cells transfected with the Trx1 over-expressing plasmid, pCMV-SPORT6 were exposed to crocidolite asbestos for the times indicated. Cells were then trypsinized and counted to estimate cell survival (*p < 0.05 compared to control; †p < 0.05 compared to Trx1 OE). Cell survival and NTB graphs are the average results of 3 experiments.

To assess ROS generation after exposure to asbestos, 90% confluent LP9 cells were exposed to 75 × 10^6^ μm^2^/cm^2^ asbestos for 24 h and incubated with NBT as described in the methods. Spectrophotometric assessment of the solubilized formazan revealed a significant increase in ROS levels when compared to controls (Figure 4A). Quantitative RT-PCR performed on cDNA from Trx1 over-expressing cells and their respective controls indicated a 4 fold increase in Trx1 levels after 48 h of transfection and this was reduced by approximately 50% 72 h post-transfection (Figure 4B). The effect of Trx1 over-expression on asbestos-induced ROS generation and cell survival was assessed by exposing 90% confluent LP9 cells transfected with a Trx1 over-expression vector (pCMV-SPORT6) and empty vector transfected controls (pcDNA) to 75 × 10^6^ μm^2^/cm^2^ crocidolite asbestos for 2 h. The levels of ROS generated in response to asbestos exposure were then measured as described previously. Cells over-expressing Trx1 were found to exhibit a trend of reduced ROS levels compared to the null controls exposed to asbestos (Figure 4C). It is to be noted here that the amount of ROS generated by asbestos after 2 h of exposure is significantly lower in magnitude than 24 h after asbestos exposure (Figure 4A). An assessment of the redox state of Trx1 in Trx1 over-expressing cells after asbestos exposure indicated that levels of reduced Trx1 were rescued in over-expressing cells as compared to control cells transfected with empty vector (EV) alone (Figure 4D). Additionally, cells over-expressing Trx1 also had increased cell survival after exposure to crocidolite asbestos (Figure 4E).

### Effect of N-acetyl-cysteine (NAC) on asbestos-induced inflammasome activation

**Figure 5:**
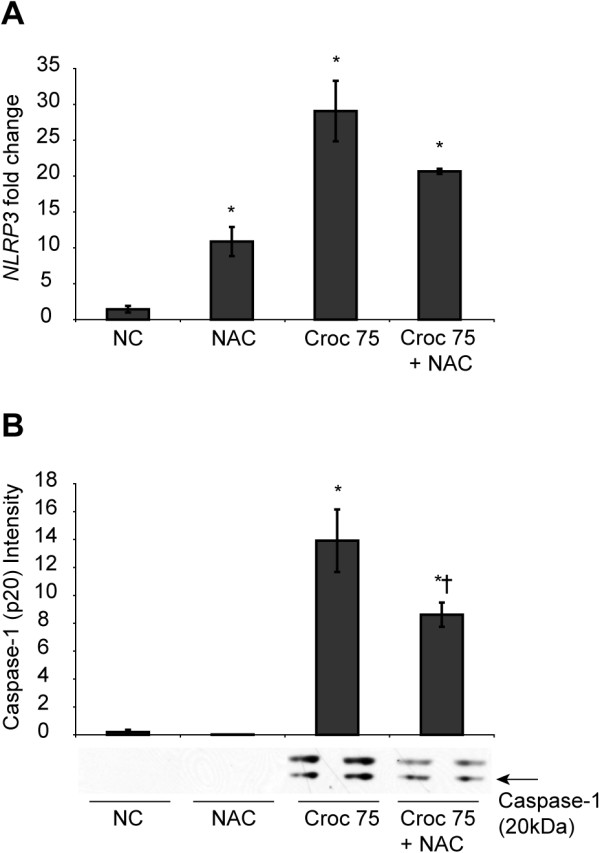
**Asbestos-induced inflammasome priming and activation is attenuated by NAC. (A)** LP9 cells pretreated with 2 mM NAC were exposed to 5 ug/cm^2^ asbestos for 48 h and changes in NLRP3 mRNA levels were assessed by qRT-PCR. **(B)** Inflammasome activation was assessed by Western blot analysis of the media supernatants from cells exposed to asbestos with and without pretreatment with NAC. Immobilized proteins on the nitrocellulose membrane were probed for the presence of active caspase-1 (p20 fragment) (*p < 0.05 compared to null control; † compared to Croc 75 alone; n = 2 per group).

Asbestos exposure results in the generation of ROS in LP9 cells, and ROS has been reported to be one of the activators of the NLRP3 inflammasome [5]. To determine whether asbestos-induced ROS plays a role in inflammasome activation, LP9 cells were pretreated with NAC and analyzed for inflammasome priming and activation by qPCR and Western blot analysis. A decrease in steady-state NLRP3 transcript levels was observed in cells pretreated with NAC prior to asbestos exposure when compared to cells exposed to asbestos alone (Figure 5A). However, this decrease in NLRP3 transcript levels with NAC pretreatment was not statistically significant. Concurrently, levels of active caspase 1 secreted into the medium after exposure of LP9 cells to asbestos were also significantly reduced after pretreatment with NAC (Figure 5B).

### TXNIP down-regulation attenuated asbestos-induced inflammasome activation

**Figure 6:**
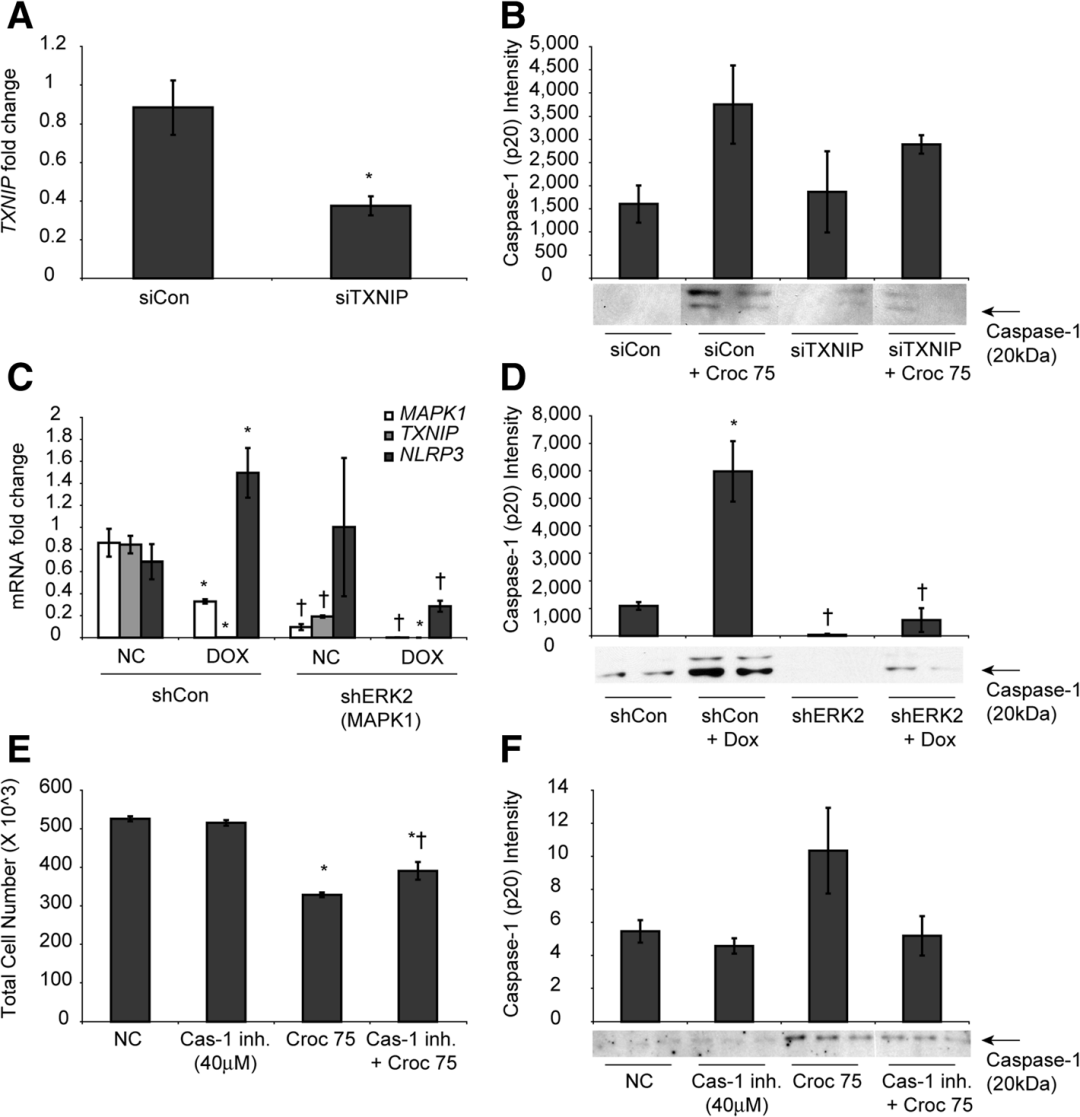
**Knockdown of TXNIP decreases inflammasome activation. (A)** LP9/hTERT cells (90% confluent) were transiently transfected with siTXNIP or siControl siRNA for 48 h and knockdown of TXNIP expression was assessed by qRT-PCR. (*p < 0.05 compared to siControl) **(B)** siControl and siTXNIP transfected cells were exposed to crocidolite asbestos for 48 h and the medium were collected, concentrated and analyzed for the presence of caspase-1 (p20) by Western blot analysis. **(C)** The transcript levels of ERK 1/2 as well as TXNIP were verified in shERK2 HMESO cells by qRT-PCR in the presence and absence of 5 μM Dox along with priming of the inflammasome (*p < 0.05 compared to shCon alone; †p < 0.05 compared to shCon + Dox; n = 2 per group). **(D)** Inflammasome activation was assessed by Western blot analysis for the p20 fragment of caspase-1 in the media supernatants after treatment with Dox (*p < 0.05 compared to shCon alone; †p < 0.05 compared to shCon + Dox; n = 2 per group). **(E)** LP9 cells were pretreated with 40 μM of the caspase-1 inhibitor VI (cas-1 inh) prior to exposure to asbestos for 48 h and cells were counted to determine survival (*p < 0.05 compared to control; †p < 0.05 compared to Croc 75 alone). **(F)** Western blot analysis for Cas-1 p20 fragment in medium supernatant from LP9 cells pretreated with the caspase-1 inhibitor prior to exposure to asbestos (n = 3 per group).

Validation of the knockdown of TXNIP expression by siTXNIP was determined by qRT-PCR and showed that an approximately 50% reduction in TXNIP mRNA levels was achieved after 48 h of transfection (Figure 6A). Western blot analysis of the cell medium after exposure to asbestos for 48 h indicated that active caspase 1 levels (caspase1-p20) were reduced after knockdown of TXNIP when compared to siControl transfected cells (Figure 6B).

In mesothelioma cell lines with a stable knockdown of extracellular signal regulated kinase 2 (shERK 2), the expression of TXNIP was found to be down-regulated 4-fold (Figure 6C). Activation of the NLRP3 inflammasome by doxorubicin treatment of shERK2 HMESO cells indicated that caspase 1 activation was drastically reduced as compared to control cells stably transfected with non-targeting shRNA (shCon) (Figure 6D) and there was no priming of NLRP3 as measured by mRNA levels of NLRP3 using qRT-PCR (Figure 6C).

Caspase-1 dependent cell death (pyroptosis), which may occur in response to inflammasome activation, may contribute to asbestos-induced cell death in addition to asbestos-induced apoptosis and lytic cell death. Therefore, we sought to determine whether pyroptotic cell death occurred in response to asbestos-induced inflammasome activation. To do so, LP9 cells were pretreated with 40 μM Caspase-1 inhibitor VI (zYVAD-FMK) before exposure to asbestos. When compared to cells exposed to asbestos alone, there was an 18% increase in cell viability, suggesting this fraction of cells may have undergone pyroptotic cell death upon exposure to asbestos (Figure 6E). Immunoblotting for Cas-1 p20 in concentrated medium supernatants also confirmed that the inhibitor attenuated activation of the caspase-1 as expected (Figure 6F).

## Discussion

Reactive oxygen species generated in response to asbestos exposure have been shown to have deleterious effects in different cell types. Asbestos-induced ROS generation has been shown to cause oxidative damage to mitochondrial and genomic DNA [21,22] and may modulate the activity/function of various signaling molecules, transcription factors and enzymes that are redox sensitive [11,21,23]. The high iron content as well as the valency state of iron on crocidolite asbestos fibers has been shown to facilitate Fenton reactions both intracellularly and extracellulary [24,25]. Mitochondrial generated superoxide can react with Fe^3+^ ions on asbestos fibers to reduce it to Fe^2+^[25]. Unfortunately, this enables the Fe^2+^ to react with any H_2_O_2_ present in the cell to produce hydroxyl radicals. This can lead to a cycle of oxidation and reduction of Fe^3+^ to produce more ROS. Additionally, the high aspect ratio of the crocidolite asbestos fibers leads to frustrated phagocytosis as mesothelial cells try to unsuccessfully phagocytose the fibers [5]. Frustrated phagocytosis promotes a sustained production of superoxide through the activity of membrane bound NADPH oxidases that are activated during the repeated phagocytosis attempts [26]. Asbestos exposure has been shown to cause a depletion of reduced glutathione levels *in vitro*[10,11], but its effects on Trx, another major antioxidant of the cell, is unknown. The ability of crocidolite asbestos fibers to activate the inflammasome in macrophages and mesothelial cells [4,5] and the involvement of the endogenous inhibitor of Trx, TXNIP, in activation of the NLRP3 inflammasome [9] led us to hypothesize that oxidation of Trx by asbestos-induced ROS may cause the dissociation of TXNIP from Trx and lead to activation of the inflammasome. Here we show for the first time that asbestos exposure of human mesothelial cells leads to oxidation of Trx and a compensatory increase in Trx1 transcript levels. We also report that TXNIP is involved in asbestos mediated activation of the inflammasome which may be as a result of the oxidation of Trx1 by asbestos-induced ROS generation.

Quantitative real time PCR showed that asbestos exposure increased the steady-state RNA levels of Trx1 by approximately 1.6 fold after 24 h. The promoter of the Trx gene (TXN) contains an antioxidant response element (ARE) [27]. It is therefore likely that ROS generated in response to asbestos exposure led to an increase in Trx1 expression as a compensatory mechanism by activating ARE-dependent gene expression. It has been reported by our group that cells exposed to asbestos show increased expression of the mitochondrial manganese superoxide dismutase (MnSOD) [11]. Studies also show that increases in expression of cellular antioxidant proteins (MnSOD and Trx1) occur in response to oxidative stress [10,11]. Exogenous thioredoxin as well as endogenously over-expressed Trx has also been shown to increase MnSOD levels in a redox dependent manner [28]. As such, increases in ROS generation in LP9 cells exposed to asbestos coupled with the oxidation of Trx1 and increased transcription of Trx1 could be considered an indication of asbestos-induced oxidative stress. To the contrary, the total intracelluar protein levels of Trx1 were reduced by about 67% in response to asbestos exposure after 24 h (Figure 1B, C). This suggests that, ROS generation by asbestos exposure may lead to an increased oxidation and removal of Trx1 protein as a conjugate, and the cells may increase transcription as a compensatory mechanism. However, Trx1 is secreted into the culture medium in response to many stimuli – it may not be degraded [29,30]. Similarly, we have demonstrated previously that asbestos exposure depletes glutathione from cells while increasing the steady-state RNA levels of γ-glutamylcysteine synthetase (a rate limiting enzyme for glutathione synthesis) at the same time [10].

To determine if asbestos exposure causes changes in the antioxidant capacity of Trx1, the oxidation state of Trx1 was assessed by redox Western blot analysis after exposure of LP9 cells to asbestos. The analysis of the oxidation states of Trx1 after asbestos exposure showed that Trx1 became irreversibly oxidized and levels of reduced Trx1 were drastically decreased compared to control cells. The diminution in the levels of reduced Trx1 may be due to the oxidation of the structural cysteine residues (Cys 62, 69 and 73). Oxidation of these structural cysteines can lead to disulfide bond formation between Cys 62 and 69 which has been shown to reduce the use of Trx1 as a substrate for TR [18]. As a result, Trx1 cannot be reduced to its active form. The formation of disulfide bonds between Cys 73 of two adjacent Trx1 molecules leading to their dimerization has also been shown to occur under extremely oxidizing conditions [31]. This dimerization may lead to conformational changes in the active site of Trx1 which will make the active site Cys residues inaccessible to TR for reduction. Other studies have shown that strong oxidants can cause the oxidation of Trx1 producing a mixture of Trx1 monomers, dimers and oligomers with no free sulfhydryl groups [32]. These findings support the possibility that asbestos-induced ROS may be involved in directly or indirectly altering the oxidation state of Trx1.

Maintenance of the reducing milieu of the cell is also important for cell survival; thus, imbalances in the ratio of cellular antioxidants and oxidants could lead to deleterious or lethal effects on the cell. To investigate what role perturbations in the redox state of the thioredoxin system plays in asbestos-induced cell death, LP9 cells were pretreated with dinitrochlorobenzne (DNCB), an irreversible alkylating inhibitor of TR [33] and assessed for cell death in response to asbestos exposure. An LDH assay as well as the Apostain assay showed that pretreatment with DNCB protected cells from asbestos induced cell death contrary to all reports in the literature in which treatment of cells with DNCB resulted in cell death [34-36]. In contrast, recent studies have shown that inhibition of TR leads to increases in GSH levels and a reduction of Trx1 and Trx2 by glutaredoxin in the absence of a functional TR enzyme [37,38]. It is therefore likely that the protection of human mesothelial cells from asbestos-induced cell death after pretreatment with DNCB may be due to increases in GSH and levels of reduced Trx1.

Levels of intracellular GSH are sensitive to ROS levels [39] and are modulated upon exposure of cells to asbestos [10,11]. Reactive oxygen species have also been shown to play a role in the activation of the NLRP3 inflammasome after asbestos exposure [6] and may be linked to the effects of GSH levels on Trx1 oxidation state and the availability of TXNIP to bind to and activate the inflammasome. To test the effect of increased GSH or reduced ROS levels on inflammasome activation, LP9 cells were incubated with 2 mM NAC for 20 h prior to asbestos exposure and this led to a significant decrease in inflammasome activation, as well as a reduction in priming. This suggests that high GSH levels may buffer reduced Trx1 levels [38] and prevent the dissociation of TXNIP from Trx1. As such, TXNIP is unable to bind to and activate the inflammasome leading to reduced levels of Caspase-1 p20 peptides in the medium supernatant. The reduction in priming of the NLRP3 may also be due to the decrease in ROS levels as GSH levels increased to buffer cells from the ROS generated in response to asbestos exposure since transcription of NLRP3 is under the control of NFκB (a redox sensitive transcription factor) [40-42]. Exposure of LP9 cells to NAC also attenuated the increase in Trx1 mRNA induced by asbestos as there was no significant increase when compared to control (data not shown). This implies that the oxidative stress induced by asbestos exposure is ameliorated by NAC.

We have demonstrated previously that crocidolite asbestos causes cell death and compensatory proliferation [3], which may be a required step for crocidolite asbestos-induced cell transformation and MM development. Our results here, indicate that a fraction of total cell death by asbestos is caused by pyroptosis (caspase-1 dependent cell death, Figure 6), a process known to be regulated in part by TXNIP. However, this observation needs to be confirmed using siRNA mediated knockdown of caspase-1 in future studies. We suspect that pyroptosis is prevented by over-expression of Trx1 (Figure 4) which renders TXNIP unavailable to subsequently induce inflammasome assembly and thus, caspase-1 activation.

Assessment of the effects of Trx1 over-expression on asbestos-induced ROS generation revealed that LP9 cells over-expressing Trx1 had lower levels of ROS after asbestos exposure when compared to vector transfected cells. Although asbestos induced a significant increase in ROS generation in LP9 cells after 24 h, the trend of reduction in ROS levels with Trx1 over-expression at an earlier time point (2 h) were not statistically significant, but reproducible. The reduction in asbestos-induced ROS generation in LP9s over-expressing Trx1 also corresponded to a moderate increase in cell survival which also exhibited a trend. Cells undergoing oxidative stress up-regulate the expression of antioxidant proteins like thioredoxin and MnSOD as well as the antioxidant peptide glutathione to counter the increase in oxidant levels [10,11,43]. As such, the reduction in asbestos-induced ROS levels upon over-expression of Trx suggests that the increase in Trx1 levels after asbestos exposure may be a compensatory mechanism to restore the antioxidant-oxidant balance that is disrupted by asbestos [21].

**Figure 7:**
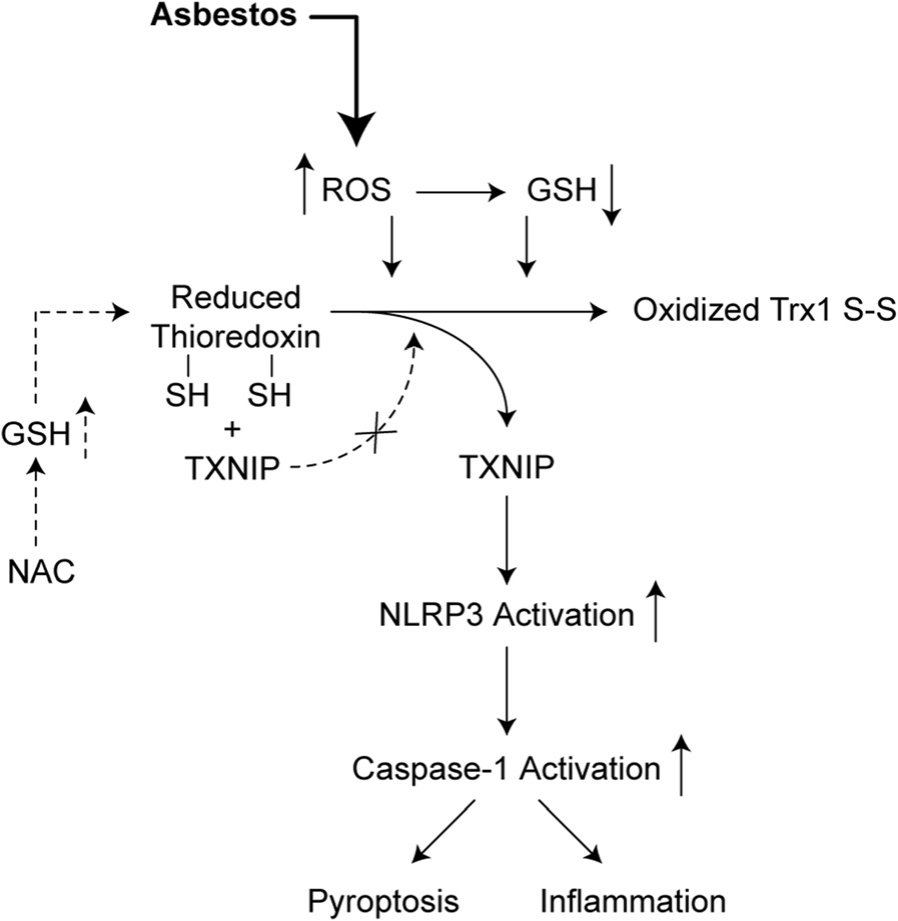
**Role of ROS and antioxidants in asbestos-induced activation of the NLRP3 inflammasome.** A simplified schema showing how increased ROS or decreased GSH as a result of asbestos exposure can cause oxidation of Trx1 and release of TXNIP. TXNIP thus released binds to NLRP3 and activates it as represented by caspase-1 activation. NAC on the other hand reduces ROS and elevates GSH levels resulting in inhibition of activation of NLRP3.

Our study also showed that the redox-dependent Trx-TXNIP interaction is involved in asbestos-induced inflammasome activation. When TXNIP, the negative regulator of Trx1 reductase activity, was knocked down in LP9 cells, inflammasome activation was reduced. Cells transfected with siTXNIP had decreased amounts of active Caspase-1 subunit p20 in the medium after exposure to asbestos when compared to control. Additionally, activation of the inflammasome by the chemotherapeutic doxorubicin in shERK2 HMESO cells, which have a four-fold lower expression of TXNIP, was attenuated, confirming that TXNIP is required for inflammasome activation. In support of our data, Zhou et al. [9] demonstrated that knockdown of TXNIP by siRNA in beta islet cells reduced activation of the NLRP3 inflammasome. Thus our findings corroborate the role of TXNIP in inflammasome activation by asbestos, and relate inflammasome activation via TXNIP to ROS levels in the cell (Figure 7).

## Conclusion

This study has demonstrated that activation of the inflammasome by asbestos is mediated in part by TXNIP as a consequence of alterations in the redox state of Trx1 in the cytosol. Further studies are in progress to offer an understanding of how asbestos-induced activation of the inflammasome and subsequent generation of biomolecules (IL-1β, IL-18, HMGB1 etc.) may lead to mesothelial cell transformation events and mesothelioma development.

## Abbreviations

TPA: 12-O-tetradecanoylphorbol-13-acetate; DNCB: 2,2-Dinitro-1-cholorbenzene; HEPES: 2-[4-(2-hydroxyethyl) piperazin-1-yl]ethanesulfonic acid; ARE: Antioxidant response element; zYVAD-fmk: Carbobenzoxy-tyrosyl-valyl-alanyl-aspartyl-[O-methyl]- fluoromethylketone; Chry: Chrysotile asbestos; Croc: Crocidolite asbestos; DHA: Dehydroascorbic acid; DMSO: Dimethyl sulfoxide; DTT: Dithiotreitol; Dox: Doxorubicin; EV: Empty vector; EDTA: Ethylenediaminetetraacetic acid; ERK2: Extracellular signal regulated kinase 2; GB: Glass beads; HBSS: Hank’s balanced salt solution; HMGB1: High mobility group box 1; LP9/hTERT (LP9): hTERT- immortalized human peritoneal cell line; HPRT: Hypoxanthine phosphoribosyl transferase; IAA: Iodoacetic acid; LDH: Lactate dehydrogenase; MM: Malignant mesothelioma; MnSOD: Manganese superoxide dismutase; NAC: N-acetylcysteine; NADPH: Nicotinamide adenine dinucleotide phosphate; NLRP3: NLR family pyrin domain containing 3; PBS: Phosphate-buffered saline; qRT-PCR/qPCR: Quantitative real time PCR; ROS: Reactive oxygen species; shERK2: Short hairpin RNA against ERK2; shCon: Short hairpin RNA non-targeting; SDS PAGE: Sodium dodecyl sulfate polyacrylamide gel electrophoresis; TBST: TBS-Tween; Trx1: Thioredoxin 1; TXNIP: Thioredoxin interacting protein; TR: Thioredoxin reductase; TBS: Tris-buffered saline.

## Competing interest

Authors declare no competing interests.

## Authors’ contributions

JT, performed experiments and wrote the manuscript with the help of AS; CW, performed NAC and inflammasome related studies; MM, performed statistical analysis and made final figures for the publication. MM also provided technical assistance with many experiments; BM, conceived the initial idea and provided intellectual input; NH, provided all the plasmids used in the study and insightful discussion; PS, helped with redox Western blots; AS, conceived the idea, designed experiments, supervised the study, interpreted data, and helped JT in writing the manuscript. All authors have read and approved the manuscript.
